# Clinical characteristics of patients with pulmonary sarcoidosis treated with systemic steroids in Japan

**DOI:** 10.3389/fmed.2025.1567334

**Published:** 2025-05-30

**Authors:** Michiru Sawahata, Hirokazu Kimura, Takeshi Hattori, Tsutomu Tamada, Masashi Bando, Makoto Maemondo, Takeshi Kawanobe, Chiyoko Kono, Tetsuo Yamaguchi, Noriharu Shijubo, Takafumi Suda, Satoshi Konno

**Affiliations:** ^1^Division of Pulmonary Medicine, Department of Medicine, Jichi Medical University, Shimotsuke, Tochigi, Japan; ^2^Department of Respiratory Medicine, Faculty of Medicine, Hokkaido University, Sapporo, Hokkaido, Japan; ^3^Department of Respiratory Medicine, National Hospital Organization Hokkaido Medical Center, Sapporo, Hokkaido, Japan; ^4^Department of Respiratory Medicine, Tohoku University Graduate School of Medicine, Sendai, Miyagi, Japan; ^5^Department of Respiratory Medicine, JR Tokyo General Hospital, Shibuya, Tokyo, Japan; ^6^Department of Respiratory Medicine, Shinjuku Tsurukame Clinic, Shibuya, Tokyo, Japan; ^7^Department of Respiratory Medicine, JR Sapporo Hospital, Sapporo, Hokkaido, Japan; ^8^Second Division, Department of Internal Medicine, Hamamatsu University School of Medicine, Hamamatsu, Shizuoka, Japan

**Keywords:** sarcoidosis, steroid, treatment, immunosuppressant, guideline

## Abstract

**Background:**

Severe pulmonary sarcoidosis is less common in Japan than in other countries, and the actual clinical situation of systemic steroid use in Japan requires clarification.

**Methods:**

This study analyzed 65 patients with histologically diagnosed sarcoidosis who initially received systemic steroids for pulmonary lesions and made regular outpatient visits to Hokkaido University Hospital, JR Sapporo Hospital, or JR Tokyo General Hospital in September 2017.

**Results:**

Median age at diagnosis was 35 (interquartile range [IQR] 26–48) years. Thirty-four patients (52.3%) were men. Median time from diagnosis to steroid initiation was 5 (IQR, 1–9) years. Median maximum steroid dose was prednisolone (PSL) 30 (range 5–60) mg/day. Immunosuppressants were used in 19 patients (29.2%). Twenty-one patients (32.3%) received PSL ≤ 10 mg/day and 7 (10.8%) received 5 mg/day. The PSL ≤ 10 mg/day group included a significantly lower proportion of men than the group treated with higher doses (33.3% vs. 61.4%, *p* = 0.034). Most cases were effectively treated, but some required long-term steroid administration. Even when steroid inhalation therapy was ineffective, systemic administration of PSL 5 mg/day effectively resolved chest imaging findings and respiratory symptoms. The successful steroid withdrawal rate was 18.5% overall, increasing to 23.8 and 42.9% in the PSL ≤ 10 mg/day and 5 mg/day groups, respectively.

**Conclusion:**

Approximately one-third of patients received an initial steroid dose of PSL ≤ 10 mg/day for pulmonary sarcoidosis. This was mostly effective and the withdrawal rate was relatively high. Our results support that some Japanese patients with pulmonary sarcoidosis may successfully receive an initial dose of PSL ≤ 10 mg/day.

## Introduction

Sarcoidosis is a condition that causes granulomatous inflammation in various organs, with the lungs being the central focus. This disease affects people regardless of age, sex, and race, and the clinical course varies widely, from spontaneous remission to cases requiring systemic steroid therapy due to exacerbation (1–3). Corticosteroids are the drugs of first choice for strongly suppressing granulomatous inflammation. However, because of the diverse clinical manifestations of sarcoidosis, the appropriate dosage of systemic steroids has yet to be adequately determined. Multiple randomized controlled trials comparing systemic steroids with placebo have reported short-term improvement in clinical, radiological, and pulmonary functional parameters in symptomatic patients (4–10). In these trials, the initial dose of prednisolone (PSL) ranged from 15 to 40 mg/day, with treatment durations ranging from 3 to 24 months (11). Furthermore, the 1999 American Thoracic Society (ATS)/European Respiratory Society (ERS)/World Association of Sarcoidosis and Other Granulomatous Disorders (WASOG) statement (12), the 2021 British Thoracic Society statement, and a Delphi consensus recommended an initial dose of PSL 20–40 mg/day (13, 14). In contrast, the 2021 ERS clinical practice guidelines recommend an initial dose of PSL 20 mg/day for the majority of patients without life-threatening conditions (15).

The SARCORT trial (16) was a physician-initiated, single-center, non-blinded, parallel-group, randomized trial that compared initial doses of PSL 40 mg/day and 20 mg/day. Both of these initial doses were demonstrated to be effective in terms of the primary outcome (suppression of relapse) and secondary outcomes (improvement in percent forced vital capacity [%FVC], health-related quality of life, and fatigue). However, PSL 40 mg/day did not show superiority in primary or secondary outcomes. Furthermore, treatment-related adverse events were prominent in patients treated with an initial dose of PSL 40 mg/day, although the difference between the two groups was not statistically significant. These results not only provide evidence that PSL 20 mg/day is a viable option for the majority of patients (ERS recommendation) (15), but also indicate the need to establish the minimum effective dose according to the affected organs and individual manifestations (16–18). Moreover, in that trial, the relapse rate after 6 months of treatment cessation was 45%, which was similar to the relapse rate after longer-term treatment in other studies. This raises the question of whether the generally recommended treatment period of 6–24 months (14, 15, 18–21) is appropriate.

In Japan, there is little evidence regarding the appropriate dose and treatment period of systemic steroids for sarcoidosis in actual clinical settings. Compared with other countries, Japan has fewer cases of severe sarcoidosis (22), and clinical practice regarding systemic steroid use needs to be clarified. We have experienced and reported cases in which low-dose steroids were effective (23). Against this backdrop, we investigated the clinical characteristics of Japanese sarcoidosis patients treated with systemic steroids, focusing on dosage, efficacy, and withdrawal rates.

## Methods

### Study population

We identified 65 patients with biopsy-proven sarcoidosis who received systemic steroids for pulmonary lesions at Hokkaido University Hospital, JR Sapporo Hospital, or JR Tokyo General Hospital during regular outpatient visits in September 2017. We excluded cases with established lung fibrosis based on the presence of traction bronchiectasis, peripheral cysts/bullae, or shrinkage of the upper lobe (24). We retrospectively reviewed the medical records and investigated the clinical situation of systemic steroid use for pulmonary lesions in actual clinical practice.

This study was reviewed and approved by the Clinical Research Ethics Committee of Hokkaido University Hospital (No. 017-0431).

### Characteristics of all patients and the low-dose steroid group

We analyzed the characteristics of all patients (*N* = 65), including sex, age at diagnosis, time from diagnosis to the initiation of steroid treatment, maximum steroid dose (PSL equivalent) at initiation, additional immunosuppressant use, and other organ involvement.

Patients who received oral PSL at a dose of ≤10 mg or 5 mg a day were classified into the low-dose steroid group. These patients were compared with those treated with higher doses, focusing on sex, age, time from diagnosis to initiation of steroid treatment, and extrathoracic involvement (e.g., eyes, skin, heart, upper respiratory tract, parotid, extrathoracic lymph nodes, gingiva, hypercalcemia). In particular, the study compared the proportion of male patients in all patients and the low-dose steroid group to investigate whether steroid doses were dependent on sex.

### Clinical course of the low-dose steroid group

The reason for steroid use (e.g., symptoms, imaging findings, etc.), efficacy, and clinical course after steroid withdrawal was analyzed in patients who received PSL 5 mg/day. Their medication history was obtained, including the history of steroid inhalation therapy.

Two respiratory specialists evaluated the patients’ chest imaging findings. These findings were classified mainly as granular/nodular shadows, ground grass opacities, or consolidations. Respiratory symptoms were classified mainly as cough or breathlessness/dyspnea.

### Steroid withdrawal rate and features of patients with successful steroid withdrawal

Successful steroid withdrawal was defined as not currently using steroids at the time of observation. The successful steroid withdrawal rate of all patients and that of the low-dose steroid group were compared to determine whether the steroid dose was associated with successful withdrawal. We also investigated the features of patients with successful steroid withdrawal. Patients with and without successful steroid withdrawal were compared, focusing on sex, age, maximum steroid dose, additional immunosuppressant use, and extrathoracic organ involvement.

### Data analysis

Continuous data are expressed as the median and interquartile range (IQR) for nonparametric data. Categorical data are presented as the absolute number and relative frequency (n, %). SYSTAT (ver. 13.2 for Windows, Systat Software, San Jose, CA, USA) was used to perform chi-square and Mann–Whitney U tests. *p* values of <0.05 were considered to indicate statistical significance.

## Results

### Patient characteristics

**Figure 1:**
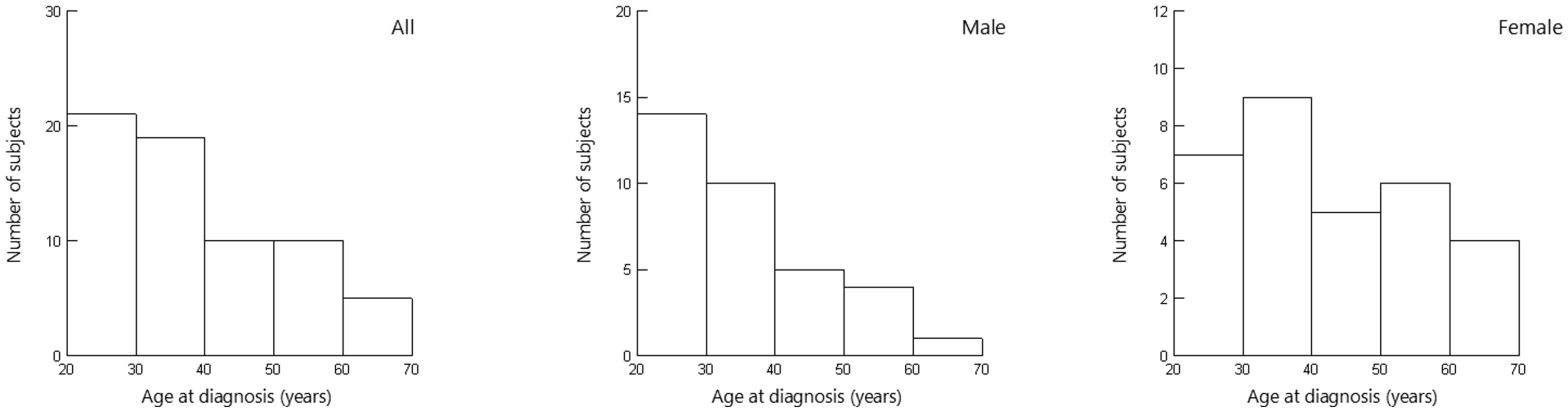
Distribution of age of sarcoidosis at diagnosis (*N* = 65). Age at diagnosis ranged widely (median, 35 [interquartile range, 26–48] years) among the 65 patients, 34 (52.3%) of whom were men.

**Figure 2:**
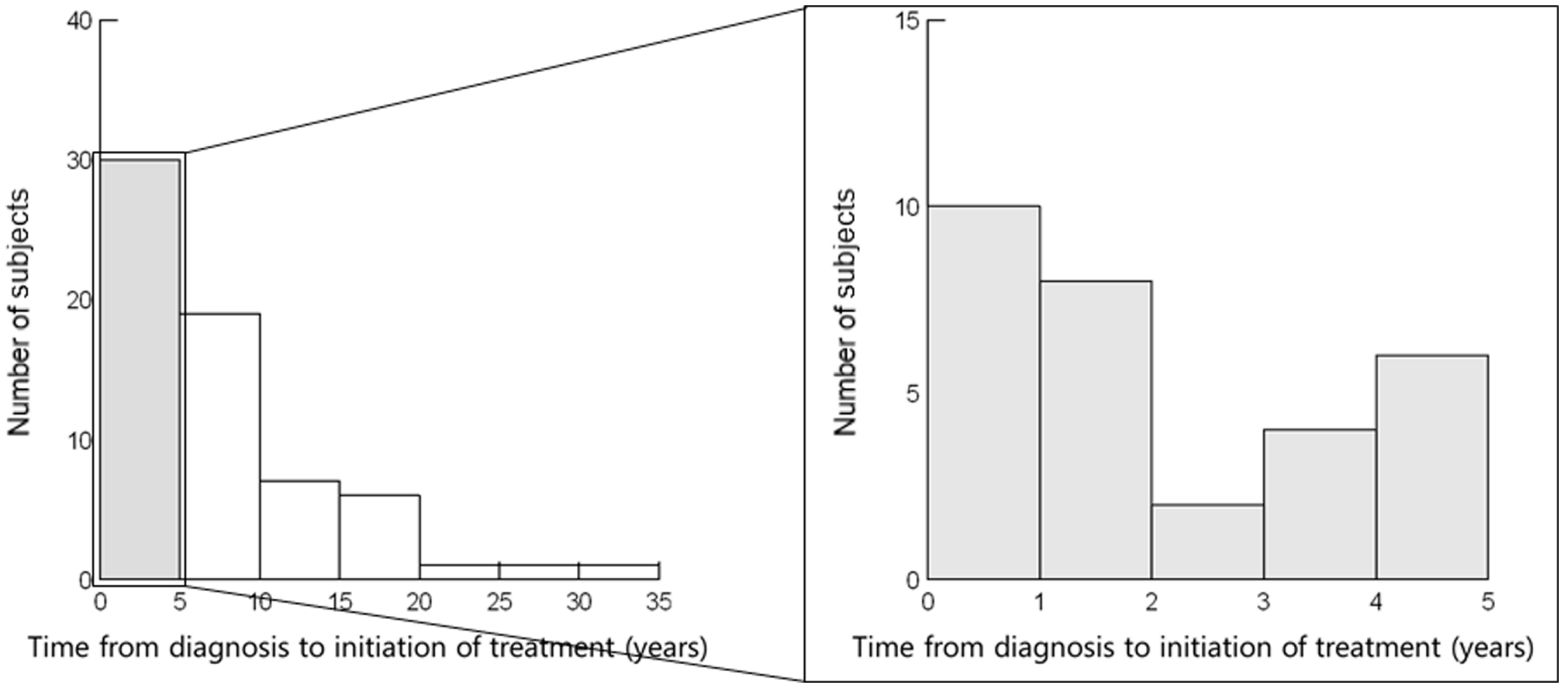
Time from diagnosis to initiation of steroid treatment (*N* = 65). Time from diagnosis to the initiation of steroid treatment varied widely (median, 5 [interquartile range, 1–9] years). Ten patients started steroid treatment within 1 year after diagnosis.

In all patients (*N* = 65), the median age at diagnosis of sarcoidosis was 35 (interquartile range [IQR] 26–48) years. Thirty-four patients (52.3%) were men (Table 1; Figure 1). The time from diagnosis to initiation of steroid treatment varied widely (median 5 [IQR, 1–9] years) (Table 1; Figure 2). Ten patients started steroid treatment within 1 year after diagnosis. The maximum PSL dose at the initiation of treatment ranged from 5 to 60 mg/day (median of 30 mg/day) (Table 1), and 19 patients received additional immunosuppressants.

**Table 1:** Patient characteristics (*N* = 65).

Sex (male/female)	34/31 (male, 52.3%)
Age at diagnosis (years)^*^	35 (26–48)
Age at initiation of steroid treatment (years)^*^	42 (36–53)
Time from diagnosis to initiation of steroid treatment (years)^*^	5 (1–9)
Maximum steroid dose (PSL equivalent, mg/day)^*^	30 (10–30)
Additional immunosuppressant use	19 (29.2)
Methotrexate only	17 (26.2)
Azathioprine only	1 (1.5)
Methotrexate and azathioprine	1 (1.5)
Other organ involvement	47 (72.4)
Ocular involvement	39 (60.0)
Cardiac involvement	5 (7.7)
Cutaneous involvement	17 (26.2)
Other	13 (20.0)

Data are presented as the number (percentage) unless otherwise indicated.

* Median (IQR).

The selection of inhaled steroid medications and the timing of their use varied among facilities and attending physicians. For instance, of the 11 cases at Hokkaido University, 7 involved the use of inhaled steroids, all during the tapering of systemic steroids, with no instances of use prior to the initiation of systemic steroids. The medications used included budesonide (BUD) 800–1,600 μg/day in 6 cases (including 1 case that switched to BUD/formoterol) and ciclesonide (CIC) in 1 case. In contrast, among 30 cases at JR Tokyo General Hospital, 18 involved the use of inhaled steroids, including at least 7 in which inhaled steroids were started before the initiation of systemic steroids. The medications used included fluticasone propionate (FP) 400–800 μg/day in 9 cases (with 1 case changing to BUD and another to CIC), FP/SM in 3 cases, BUD in 2 cases (with 1 case changing to FP/salmeterol), vilanterol/fluticasone furoate in 2 cases, CIC in 1 case, and an unknown steroid in 1 case.

Forty-seven patients (72.4%) had other organ involvement (e.g., eyes, skin, or heart).

### Features of the low-dose steroid group

PSL was administered at a dose of ≤10 mg/day in 21 patients (32.3%) and 5 mg/day in 7 patients (10.8%). The median age at diagnosis of sarcoidosis in the ≤10 mg/day and 5 mg/day groups was, respectively, 36 (IQR, 26–49) years (Table 2) and 30 (IQR, 24–46) years (Table 3), respectively. The median time from diagnosis to initiation of steroid treatment in the respective groups was 7 (IQR, 3–11) years and 8 (IQR, 4–13) years. Fifteen (71.5%) of the 21 patients in the PSL ≤ 10 mg/day group and 5 (71.4%) of the 7 patients in the 5 mg/day group had other organ involvement.

**Table 2:** Comparison of the overall cohort and the PSL ≤ 10 mg/day and >10 mg/day groups.

	Overall	PSL ≤ 10 mg	PSL > 10 mg	*p* value
	(*N* = 65)	(*n* = 21)	(*n* = 44)	
Sex (male/female)	34/31	7/14 (male, 33.3%)	27/17 (male, 61.4%)	0.034
Age at diagnosis (years)^*^	35 (26–48)	36 (26–49)	34 (26–46)	0.598
Age at initiation of steroid treatment (years)^*^	42 (36–53)	44 (39–57)	41 (36–50)	0.135
Time from diagnosis to initiation of steroid treatment (years)^*^	5 (1–9)	7 (3–11)	5 (1–9)	0.226
Extrathoracic involvement	47 (72.3)	15 (71.5)	32 (72.7)	0.913
Cardiac involvement	5 (7.7)	4 (9.1)	1 (4.7)	0.540
Neurologic involvement	2 (3.1)	1 (4.8)	1 (4.7)	0.587
Steroid withdrawn	12 (18.5)	5 (23.8)	7 (15.9)	0.526

PSL: prednisolone.

Data are presented as the number (percentage) unless otherwise indicated. ^*^Median (IQR).

**Table 3:** Comparison of the overall cohort and the PSL 5 mg/day and PSL > 5 mg/day groups.

	Overall	PSL 5 mg	PSL > 5 mg	*p* value
	(*N* = 65)	(*n* = 7)	(*n* = 58)	
Sex (male/female)	34/31	2/5 (male, 28.6%)	32/26 (male, 55.2%)	0.183
Age at diagnosis (years)^*^	35 (26–48)	30 (24–46)	36 (26–48)	0.427
Age at initiation of steroid treatment (years)^*^	42 (36–53)	38 (33–56)	42 (36–52)	0.874
Time from diagnosis to initiation of steroid treatment (years)^*^	5 (1–9)	8 (4–13)	5 (1–9)	0.214
Extrathoracic involvement	47 (72.3)	5 (71.4)	42 (72.4)	0.956
Cardiac involvement	5 (7.7)	0 (0)	5 (8.6)	0.419
Neurologic involvement	2 (3.1)	0 (0)	2 (3.4)	0.618
Steroid withdrawn	12 (18.5)	3 (42.9)	9 (15.5)	0.078

PSL: prednisolone.

Data are presented as the number (percentage) unless otherwise indicated. ^*^Median (IQR).

Thirty-four of the 65 patients (52.3%) in this study were male (Table 1). However, this proportion decreased to 33.3% (7 of 21) in the ≤10 mg/day group (Table 2) and 28.6% (2 of 7) in the 5 mg/day group (Table 3).

The comparison of patients treated with low-dose PSL (PSL ≤ 10 mg/day or PSL 5 mg/day) and those treated with higher doses, focusing on age at diagnosis, age at initiation of steroid treatment, time from diagnosis to initiation of steroid treatment, and extrathoracic involvement, revealed no significant differences (Tables 2, 3). However, the proportion of male patients was significantly lower in the ≤10 mg/day group than in the higher dose group (33.3% vs. 61.4%, *p* = 0.034).

### Clinical course after steroid withdrawal in the PSL 5 mg/day group

The clinical courses of the 7 patients who received PSL 5 mg/day are shown in Table 4. Five of the patients were female, 3 were in their 20s, and 5 had bilateral hilar-mediastinal lymphadenopathy. Steroid therapy was initiated at a median of 8 (IQR, 4–13) years after diagnosis (Table 3). The main reason for steroid use in these 7 patients was worsening of lung involvement (indicated mainly by granular/nodular shadows on radiographic images and respiratory symptoms such as breathlessness and coughing).

**Table 4:** Details of eight patients who received PSL 5 mg for lung involvement.

Sex	Age at diagnosis	Lung involvements (Reasons for steroid use)	Other organ involvement	Time from diagnosis to steroid initiation (years)	Effectiveness of systemic steroids	Steroid withdrawal /continuation
F	60	Granular and nodular shadows, consolidations, BHL (Ineffective steroid inhalation therapy)		8	Effective	Successfully withdrawn
F	30	Granular and nodular shadows, consolidations (Breathlessness, ineffective steroid inhalation therapy)		1	Effective	Successfully withdrawn
F	23	Granular and nodular shadows, BHL (Coughing, increases in shadows, ineffective steroid inhalation therapy)	Eye	8	Effective (requiring ≥4 months to improve)	Successfully withdrawn
F	25	Granular shadows, calcification of mediastinal lymph nodes (Increases in shadows)	Eye, skin, gingiva, upper respiratory tract	30	Effective	Continued (due to worsening when steroids were withdrawn)
M	23	Granular shadows, ground grass opacities, cysts, BHL (Coughing and breathlessness, increases in shadows, ineffective steroid inhalation therapy)	Eye, skin Parotid, Extrathoracic lymph node	15	Effective	Continued (due to worsening when steroids were withdrawn)
F	49	Granular and nodular shadows, BHL (Increases in shadows)	Eye, skin	7	Effective	Continued (due to worsening when steroids were withdrawn)
M	35	Granular shadows, BHL (Increases in shadows, ineffective steroid inhalation therapy)	Eye, hypercalcemia	3	Effective^*^	Continued (due to worsening when steroids were withdrawn)

BHL: bilateral hilar-mediastinal lymphadenopathy.

* Treated with methotrexate.

Systemic steroid administration was effective in all 7 patients, including 5 patients for whom steroid inhalation therapy was ineffective. However, the shadows on radiographic images disappeared only after long-term steroid use (≥4 months) in 1 patient. Steroids could be withdrawn during the follow-up period in 3 patients, all of whom were female. However, steroids were resumed after withdrawal in the remaining 4 patients due to worsening lung involvement on radiological imaging. All 4 of these patients had multiple organ involvement (e.g., eyes or skin), in addition to pulmonary involvement.

### Features of patients with successful steroid withdrawal

During a median observation period of 7.5 (range, 0–30) years after the initiation of steroids, successful withdrawal from steroid treatment was achieved by 12 (18.5%) of the 65 sarcoidosis patients (Table 5). This rate increased to 23.8% (5/21) in the PSL ≤ 10 mg/day group (Table 2) and 42.9% (3/7) in the 5 mg/day group (Table 3). The 12 patients in whom successful withdrawal was achieved included 7 women and 10 patients managed without additional immunosuppressants.

**Table 5:** Characteristics of patients with and without successful steroid withdrawal (*N* = 65).

	Steroids withdrawn	Steroids continued	*p* value
	(*n* = 12)	(*n* = 53)	
Sex (male/female)	5/7 (male, 41.7)	29/24 (male, 54.7)	0.414
Age at diagnosis (years)^*^	35 (23–52)	35 (26–47)	0.703
Age at initiation of steroid treatment (years)^*^	39 (31–52)	42 (38–54)	0.314
Maximum steroid dose (PSL equivalent, mg/day)^*^	25 (7.5–30)	30 (10–30)	0.792
Immunosuppressant use	2 (16.7)	17 (32.1)	0.289
Other organ involvement	7 (58.3)	40 (75.5)	0.231
Ocular involvement	6 (50.0)	33 (62.3)	0.434
Cardiac involvement	2 (16.7)	3 (5.7)	0.196
Cutaneous involvement	2 (16.7)	15 (28.3)	0.408

PSL: prednisolone.

Data are presented as the number (percentage) unless otherwise indicated.

* Median (IQR).

Chi-square or Mann–Whitney U tests were performed, as appropriate.

We also demonstrated that steroid withdrawal tended to be achieved more frequently in patients treated with PSL 5 mg/day relative to those treated with higher doses (42.9% vs. 15.5%, *p* = 0.078) (Table 3). In addition, steroid withdrawal tended to be achieved more frequently by female patients than by male patients (58.3% vs. 45.3%, *p* = 0.414) (Table 5). Finally, steroid withdrawal tended to be achieved more frequently in patients managed without additional immunosuppressant than in those managed with additional immunosuppressants (83.3% vs. 67.9%, respectively, *p* = 0.289). These groups showed no significant differences in age at diagnosis, age at initiation of steroid treatment, maximum steroid dose, or extrathoracic organ involvement.

## Discussion

This is the first study to investigate the clinical situation of systemic steroid use for pulmonary lesions in actual clinical practice in Japan, with a focus on efficacy and the steroid withdrawal rate among patients initially treated with low-dose steroids. Four important findings were obtained. First, the 65 Japanese sarcoidosis patients who were treated with different doses of systemic steroids showed diverse clinical features and treatment courses. Age at diagnosis ranged widely (median, 35 [IQR, 26–48] years), 34 (52.3%) of the patients were men, and the time from diagnosis to initiation of steroids varied widely (median, 5 [IQR, 1–9] years). Second, the maximum steroid dose ranged from PSL 5 to 60 mg/day (median, 30 mg/day). Nineteen patients were managed with additional immunosuppressants. Twenty-one patients (32.3%) received PSL ≤ 10 mg/day, while 7 (10.8%) received PSL 5 mg/day. The PSL ≤ 10 mg/day group included a significantly lower proportion of male patients relative to patients who received higher doses (33.3% vs. 61.4%, *p* = 0.034). Third, in most cases, patients who received PSL 5 mg/day were effectively treated, but these patients sometimes required long-term administration. Even in cases where steroid inhalation therapy was ineffective, systemic steroid administration of PSL 5 mg/day was effective for relieving chest imaging findings and respiratory symptoms. Fourth, the successful steroid withdrawal rate of 18.5% (12 of 65) increased to 23.8% in the PSL ≤ 10 mg/day group and 42.9% in the 5 mg/day group.

The maximum PSL dose at the initiation of treatment among the subjects ranged widely from 5 to 60 mg/day (median, 30 mg/day). Japan was recognized as having a lower incidence of severe pulmonary sarcoidosis relative to other countries; however, there is a dearth of Japanese studies that specifically focus on the actual treatment of sarcoidosis. In Japan, prior to around 2017, the “Views on the treatment of sarcoidosis-2003 (25)” were widely used, and PSL 30 mg/day or 60 mg every other day was recommended for sarcoidosis-related pulmonary involvement. However, we showed that, in Japan, approximately one-third of patients treated with systemic steroids for pulmonary involvement received PSL at an initial dose of ≤10 mg/day. Our findings suggest that Japanese patients diagnosed with pulmonary sarcoidosis may be able to tolerate an initial daily dose of PSL ≤ 10 mg. It should be noted that a daily dose of 5 mg was often effective (as determined by chest imaging findings and respiratory symptoms), even in cases where steroid inhalation therapy was not effective.

As for global trends, the SARCORT trial confirmed that PSL 20 mg/day and 40 mg/day were effective in terms of the primary (relapse suppression) and secondary (%FVC and improvement in health-related quality of life and fatigue) outcomes (16). While the superiority of PSL 40 mg/day was not demonstrated in either the primary or secondary outcomes, in the PSL 40 mg/day group, treatment-related adverse events were slightly more prominent. Therefore, it was concluded that the minimum effective dose needs to be determined with consideration of individual severity and organ involvement (17, 18). According to the ERS clinical practice guidelines (15), an initial dose of 20 mg/day is recommended, followed by 5–10 mg each day to every other day. Many treatment guidelines set the target maintenance dose at PSL < 10 mg/day (26).

In Japan, the release of “The Japanese Society of Sarcoidosis and Other Granulomatous Disorders (JSSOG) Diagnostic Standard and Guideline for Sarcoidosis-2020,” which recommended a dosage of PSL 0.5 mg/kg/day, was succeeded by the release of “The JSSOG Diagnostic Standard and Guideline for Sarcoidosis-2023” (27), which established an initial dose of PSL 20–30 mg/day as the standard treatment for lung involvement. The latter guideline allows for the use of lower doses, with the option of PSL 5–10 mg/day. When tapering is difficult or the response is poor, the use of methotrexate (MTX) at a dose of 6–8 mg/week in combination with PSL is recommended, and there is also an option to use the anti-fibrotic agent nintedanib. Further evidence is needed on the effects of systemic steroids at various doses and their combination with additional immunosuppressants, including MTX and anti-fibrotic agents, in the treatment of sarcoidosis-related pulmonary involvement.

It was not the steroid dose itself that enabled successful steroid withdrawal but the individual’s low steroid requirement based on disease severity. Steroid withdrawal tended to be easily achieved in patients who were initially treated with low-dose steroids and those managed without additional immunosuppressants. This is supported by the discussion in the SARCORT trial, which reported that “the tendency to relapse after treatment completion is probably host-dependent and disease-specific rather than being dependent on the treatment intensity and duration” (16). In this trial, the relapse rate among patients who completed 6 months of treatment was 45%, and this rate was comparable to that observed after longer-term treatment in other studies (5, 15, 28–30). Sarcoidosis is considered to be an amplified and persistent granulomatous reaction to inhaled causative antigens such as *Cutibacterium acnes* and *Mycobacterium*, and the long-term use of low-dose steroids may effectively maintain remission by relieving the underlying disequilibrium between the Th1 and Th17 response and regulatory mechanisms against such antigens (31–33).

Combining the first and second observations, steroid therapy was initiated more frequently and was administered at higher doses for pulmonary sarcoidosis in men compared with women. We consider that sex could potentially impact the initiation and dosage of steroid therapy. The proportion of males among the 65 sarcoidosis patients in this study was 52.3%, while this proportion among 4,838 sarcoidosis patients with pulmonary involvement, who newly applied for medical expense subsidy application between 2002 and 2011, was recorded to be 42.7% in our previous nationwide survey (34). Previous epidemiological studies (35, 36) have noted that men with sarcoidosis are more likely to have lung involvement than women, which may partly explain the high proportion of males who received systemic steroid treatment in this study. Moreover, young male patients with pulmonary involvement may be more likely to use systemic steroids. The median age at diagnosis in the 65 patients in this study was 35 years, whereas that in our previous nationwide survey of 4,838 patients was 51 years (33). Age ≤45 years at diagnosis, as well as being Black, and having multiple organ involvement, were identified as independent predictors for initiating PSL treatment in a large multicenter study with 1,445 sarcoidosis patients from 10 countries, including Japan (22). Another possibility is that women may tend to be anxious about the toxicity of corticosteroid treatment due to potential side effects, which may influence the treatment decisions of individual physicians.

The strength of this study lies in evaluating 65 patients with pulmonary sarcoidosis requiring systemic steroids across 3 referral hospitals in Japan, where such severe cases are relatively rare. However, this study has significant limitations that must be taken into consideration when interpreting the results. First, this study was entirely descriptive and retrospective in nature. This limited number of patients may hinder the generalizability of the findings, and the severity and diversity of the cases in this study might not be representative of those in actual clinical practice. Second, and most importantly, this study was limited by some potential sources of selection bias. Although treatment decisions for all patients were made by pulmonary physicians with expertise in the management of sarcoidosis, the decisions regarding the initiation of steroids and the determination of the dosage were made entirely at the discretion of the individual physicians. Therefore, we cannot definitively conclude that low-dose steroid treatment is an appropriate choice for selected patients with pulmonary sarcoidosis. The successful withdrawal of steroids appeared to be attributable to the individual’s low steroid requirement, which was based on the severity of their disease. Steroid withdrawal rates were influenced by observation periods. It is recommended to examine the rationale for using low-dose steroids based on information about disease severity based on the Scadding criteria and pulmonary function tests, with sufficient observation periods. Consequently, we are planning larger, multicenter studies to utilize this information on disease severity.

In conclusion, in actual clinical settings in Japan, approximately one-third of patients treated with systemic steroids for pulmonary sarcoidosis received PSL at an initial dose of ≤10 mg/day. In most cases, this was considered to be effective based on chest imaging findings and respiratory symptoms, even in cases in which steroid inhalation therapy was ineffective. Some patients required long-term administration; however, their relapse rate after steroid withdrawal was relatively low, probably reflecting the low steroid requirement of these individuals due to mild disease severity. Our results provide evidence to support that some patients with pulmonary sarcoidosis in Japan may be successfully treated with PSL at an initial dose of ≤10 mg/day. There is a need to establish the minimum effective steroid dose specific to Japan, where severe cases of pulmonary sarcoidosis are relatively rare.

## Data Availability

The raw data supporting the conclusions of this article will be made available by the authors without undue reservation.
